# The aspiration for scalable citizen-level interventions and the missing tests of countermessaging

**DOI:** 10.1073/pnas.2527948123

**Published:** 2026-01-12

**Authors:** Ariel Malka, James N. Druckman

**Affiliations:** ^a^Departments of Psychology and Political Science, Yeshiva University, New York, NY 10033; ^b^Department of Political Science, University of Rochester, Rochester, NY 14627

A surge of recent research has tested the efficacy of citizen-level interventions to reduce partisan animosity and antidemocratic sentiment in the United States (1–4). Brief, inexpensive approaches are appealing because they could plausibly be implemented at scale. Yet concerns remain about the durability of their effects once citizens return to an information environment saturated with partisan messaging.

In a recent PNAS paper, Hall et al. [*Durably Reducing Partisan Animosity Through Multiple Scalable Treatments* (5)] address this issue by examining whether bundling interventions produces more persistent outcomes. The researchers exposed respondents to three weekly videos intended to reduce animosity (e.g., a video of Democrats and Republicans interacting positively) and found significant reductions that persisted, at diminished levels, 2 to 4 wk later. They argued that because these interventions are brief and easy to administer, their results provide a basis for large-scale deployment. Their results contrast with another recent PNAS paper by Holliday et al. (6) that shows the effects of interventions mostly decaying after 2 wk, with small surviving effects failing to translate into reduced antidemocratic attitudes. This suggests that without institutional reforms that alter elite incentives to promote polarization, behavioral interventions are unlikely to have societal consequences.

We applaud the careful assessments of durability and the value of bundling interventions. But we also encourage researchers designing potentially scalable interventions to incorporate realistic countermessaging into their designs. Measuring outcomes weeks later shows whether effects endure under ordinary political conditions. Yet a sizeable rollout involving an initiative associated with politicians or nonprofits would itself be likely to alter the political information environment. In one scenario, interventions might be widely publicized to stimulate broad attention and uptake; in another, they might be embedded within antibias training programs. In either case, as scalability increases so might attention from elites who have incentives to cultivate animosity and, in some cases, misinform the public about their own or their party’s antidemocratic behavior. Such actors would likely mobilize countermessages aiming to preempt or undermine the intervention.

Prior research illustrates this vulnerability. Correcting misperceptions of outpartisans’ willingness to engage in antidemocratic behavior and partisan violence reduces partisans’ own support for such behavior (2, 4). Yet exposure to a simple, realistic countermessage—either questioning the evidence or promoting a counternarrative—eliminated the effect (7).

Hall et al., like many studies in this area, address one dimension relevant to scalability: time. Future work gauging the effectiveness of citizen-level interventions would benefit from considering another aspect of contextual validity: How implementation might shape the broader information environment. Incorporating realistic countermessages into experimental designs may not only reveal vulnerabilities a la Holliday et al. but also uncover opportunities a la Hall et al. For instance, iterative competing communications might in some cases prompt greater cognitive elaboration among citizens (8), which could potentially yield downstream benefits. Our point is to encourage greater attention to the practical consequences of scaling up (9) and to the dynamics of message competition when designing interventions.
